# Investigation into Co and Ga_2_O_3_ co-doped ZnSe chalcogenide composite semiconductor thin films fabricated using PLD

**DOI:** 10.1039/c8ra02466a

**Published:** 2018-04-19

**Authors:** Yong Pan, Li Wang, XuQiong Su, ShuFeng Li, DongWen Gao, XiaoWei Han, HuanHuan Yan

**Affiliations:** College of Applied Sciences, Beijing University of Technology Beijing 100124 China lwang.1@bjut.edu.cn +86-010-67392198

## Abstract

(Ga_2_O_3_)_0.1_(Co)_0.5_(ZnSe)_0.4_ thin films were fabricated *via* PLD at different pressures and substrate temperatures. The influence of different preparation conditions on the thin films was deeply explored through investigating the structural, optical and electromagnetic properties, and surface morphologies. The thicknesses of the thin films were greatly affected by the preparation conditions. The poor light transmittance of the thin films under conditions of 4 Pa and 600 °C was revealed through refractive index measurements. The stable amorphous structure was confirmed *via* XRD. The optimum preparation conditions, room temperature, 800 °C and 10 Pa, were reflected in the transmission spectra. Greater energy transfer between each of the energy levels and more activity under the temperature conditions used were indicated through PL spectra. The lower resistivity and higher carrier concentration in the quartz substrate were shown in the results of Hall effect measurements. The significant impact of high temperature preparation conditions on the thin films was visualised using AFM. All of the results indicated that the properties of the thin films are significantly influenced by the preparation conditions. Furthermore, a semiconductor chalcogenide material with excellent optical and electromagnetic properties was proposed in this investigation.

## Abstract 2

(Ga_2_O_3_)_0.1_(Co)_0.5_(ZnSe)_0.4_ thin films were fabricated *via* PLD at different pressures and substrate temperatures.

## Graphical abstract

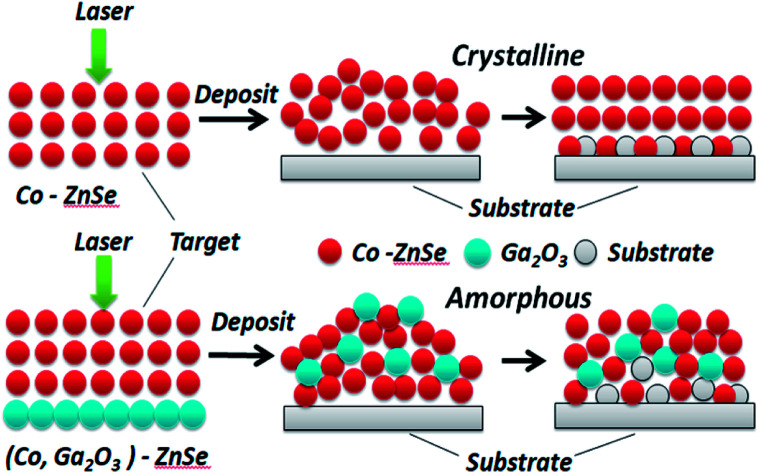

## 1. Introduction

ZnS/Se chalcogenide materials have been applied to optical sensing, testing, manufacturing and other fields because of their advantages of fast optical response times, low optical losses, and low phonon energies.^1–4^ These excellent properties in materials can be obtained *via* a combination of different doping ions, especially through the method of co-doping.^5–8^ Hence, great significance is placed on chalcogenide based composites. Transition metal (TM) ions, such as Fe, Ni, Co, Cr, Mn, are considered as the most potentially active ions for the preparation of infrared lasers, because of their abundant emission levels in the infrared region.^9–13^ Among most TM ions, the enhancement and expansion of the optical properties of chalcogenide materials in the infrared region can be achieved through introducing Co elements, due to their abundant absorption and emission levels and magnetic properties.^14,15^ Meanwhile, the electrical and luminescence properties of Ga_2_O_3_, a kind of wide band gap semiconductor (4.9 eV) material, have attracted the most attention for a long time. An improvement in photoelectric properties can be realized by doping Ga_2_O_3_.^16^ Therefore, various doping methods have been deeply studied, such as (Co, Ga)–ZnO,^17,18^ (Co, Fe)–ZnS,^19^ Co–ZnSe,^20,21^ (Cr, Ni, Co. Ti)–ZnSe,^22^ Ga_2_O_3_–In_2_O_3_,^23^ Co–Ga alloys,^24^ and (Co, Ni)–ZnS.^25^ In terms of the form of materials, four patterns have been focused on in research: ceramic,^26,27^ thin film,^28,29^ nanoparticle^30^ and quantum dot.^31^ The structures, and ferromagnetic and optical properties of doped materials have been mainly investigated. However, there are still many unsolved problems. Firstly, co-doped ions often exist in the form of elements with relatively similar properties, such as Co and Fe. Then, materials studies into various patterns are not comprehensive enough to obtain full research understanding. Finally, the problems of single doping methods, application scopes that are too small, and no substantial breakthroughs have become the main concerns of researchers. Furthermore, the effects of the different performances of thin films and optimization strategies should be discussed. In fact, in order to solve the above problems, it is necessary to make deeper excavations into material investigations, with a wider range of performance studies, and more innovative ways of doping.

Many techniques have been used to prepare thin films, such as pulsed laser deposition (PLD),^32^ molecular beam epitaxy (MBE),^33^ and metal organic chemical vapor deposition (MOCVD).^34^ The plasma transport mechanism of PLD determines that films deposited *via* this technology are consistent with the target components, which is especially suitable for complex components and high melting point films. As such, we sought to employ this method to transfer TM structures and embed them into chalcogenide materials.

In previous work,^35^ a material with Co and Ga_2_O_3_ co-doped into a ZnS/Se ceramic was reported for the first time. In this paper, thin films with Co and Ga_2_O_3_ co-doped into ZnSe are fabricated *via* PLD, using the ceramic bulk as the target. The differences between films grown on the two substrates of sapphire and quartz are deliberated. The effects of different preparation conditions on the properties of thin films are studied. So we examined the effects of temperature and pressure on the process of film growth. Besides, this work is a bridge between nano-materials and their source materials, and also a link between research into different morphological materials, which has a certain significance. The aim of our investigation is to obtain the optimum preparation conditions and experimental parameters for (Co, Ga_2_O_3_)–ZnSe thin films, and to achieve a wide range of materials with excellent photoelectric properties. The thin films can be applied to two areas. On one hand, they can be used in solar cells, field effect devices, threshold switches and memory switching devices. On the other hand, these films are the basis for the preparation of chalcogenide glass.

## 2. Experimental

(Ga_2_O_3_)_0.1_(Co)_0.5_(ZnSe)_0.4_ thin films was prepared *via* PLD, using ceramic bulk materials, which were fabricated according to previous research. Quartz (SiO_2_) and sapphire (Al_2_O_3_) substrates were selected for this experiment. The crystalline quality and electrical properties of the films are greatly affected by the substrate type and its surface cleanliness. So all of the substrates were placed in acetone, alcohol and deionized water in turn and cleaned for 10 minutes *via* an ultrasonic cleaning machine. Then the substrate was placed in the middle of a substrate tray and put into a vacuum chamber. The vacuum pressure in the chamber was adjusted to less than 10 Pa through a mechanical pump and then to less than 3 × 10^−4^ Pa using a molecular pump. A laser was focused on the target at a 45 degree angle through a lens with a focal length of 60 cm. The average power of the laser was adjusted to 400 mW, and the deposition time was set to 30 min.

In order to understand the different effects of different preparation conditions and experimental environments on the performance of the films, the pressure in the vacuum chamber was changed in the range of 2–10 Pa, using Ar gas, and the substrate temperature was raised from room temperature (RT) to 800 °C, using a substrate heating device installed in the chamber. When the temperature was changed, the pressure was maintained as normal; as the temperature remained at RT, the pressure was changed.

A frequency-tripled 355 nm Q-switch Nd:YAG laser (GCR-170, Spectra Physics) with a pulse duration of 10 ns, a repetition rate of 10 Hz, and a pulse energy of 30 mJ was used in the fabrication. Any impurity gas in the vacuum chamber was monitored *via* a mass spectrometer (USB-NS-PYRO1915). The thickness and refractive index were measured *via* a thin-film analyzer (Filmetrics F20). The structures of the (Ga_2_O_3_)_0.1_(Co)_0.5_(ZnSe)_0.4_ thin films were analyzed through XRD (BRUKER D8 ADVANCE). The transmission spectra of the thin films were examined using UV-vis and near-infrared spectra, obtained using a U-4100 spectrometer. PL spectra of the thin films were collected using a spectrometer (Ocean Optics NIR512&S2000). The electrical properties were tested using a Hall-effect measurement system (Eastchanging ET 9000). The surface morphologies of the thin films were imaged *via* AFM (JEOL JSM 6500F).

## 3. Results and discussion

### 3.1. Chamber atmosphere and basic properties

**Fig. 1:**
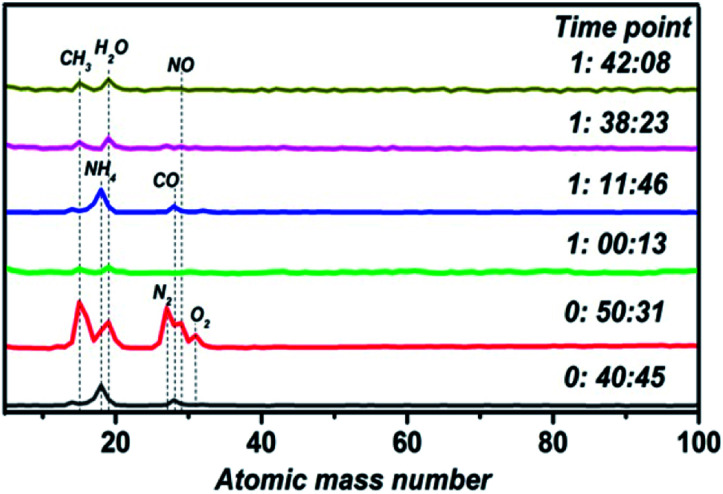
Vacuum chamber atmospheric monitoring results show that the mass loss rate depends on the sintering temperature.

Vacuum chamber atmospheric monitoring during the process of film fabrication is shown in Fig. 1. There are two processes resulting in the formation of impurities. Firstly, the door of the vacuum chamber was opened to replace the sample and substrate before each experiment, so an amount of impurity gas entered the vacuum chamber. Besides, many experiments with different materials have been conducted in the vacuum chamber, which leaves a lot of impurities in the chamber. At the time point of 0 : 40 : 45, NH_4_ and CO exist in the vacuum chamber with an unopened molecular pump. The state of the molecular pump is changed to being fully open 10 minutes later, so many impurities are pushed into the chamber, including CH_3_, H_2_O, N_2_ and O_2_. Then, there is a sharp decrease in impurity gas concentration after working the molecular pump for ten minutes. By the time point of 1 : 11 : 46, the molecular pump is closed and the preparation of the thin film begins. Therefore, impurity gas containing NH_4_ and CO returns, increasing in concentration upon the closing of the molecular pump valve gate. With the progression of thin film preparation, the last two time points show that impurities in the vacuum chamber are gradually reduced. Hence, a stable atmospheric environment is maintained during the process of thin film preparation.

**Fig. 2:**
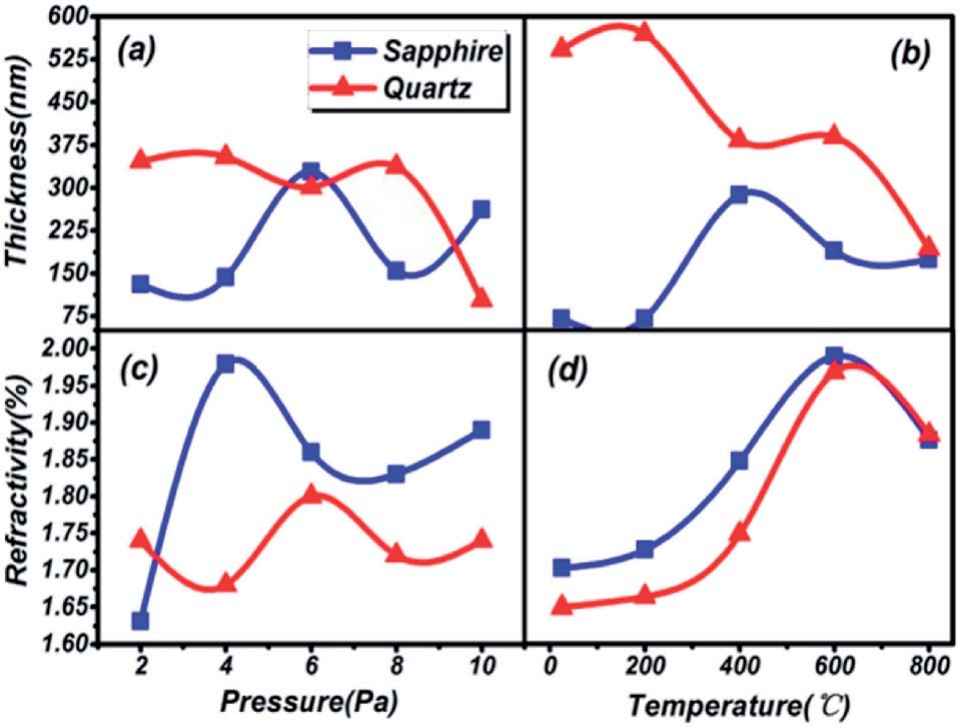
The thickness and refractivity relationships depend on the pressure and substrate temperature in the vacuum chamber on different substrates. The sapphire substrate and quartz substrate are represented by blue squares and red triangles, respectively.

The thickness and refractivity relationships depend on the pressure and temperature in the vacuum chamber on different substrates, as shown in Fig. 2. Opposing trends in thickness appear in both Fig. 2(a) and (b). The thicknesses of thin films on a quartz substrate are higher than on a sapphire substrate; meanwhile, the temperature dependence is greater than the pressure dependence. However, the film thickness on the quartz substrate is inclined to decrease on the whole. The thickest film is 571.08 nm at 200 °C and the thinnest film is 100.59 nm at 10 Pa, on the quartz substrate. Furthermore, the peak thicknesses for thin films on sapphire are located at 6 Pa and 400 °C, and the lowest thickness, 52.62 nm, is found in Fig. 2(b), reaching the level of nanoparticles. It can be considered that the thicknesses of thin films are greatly influenced by the preparation conditions. Besides, the same trends in refractive index are depicted in Fig. 2(c) and (d). The variations in the curves are similar to a sinusoidal trend, and this rule is closely followed for films prepared under conditions of changing pressure and substrate temperature. The maximum index of refraction values are 2.00 on the sapphire substrate and 1.97 on the quartz substrate under preparation conditions of 600 °C, and 1.98 on the sapphire substrate at 4 Pa. A larger refractive index indicates that the material is a denser medium. What's more, a higher refractive index means that light from one side of the material is more prone to total reflection at the other side of the material when there is a boundary with a low refractive index medium (such as air).^36^ Therefore, poor light transmittance by the thin films is identified as occurring under conditions of 4 Pa and 600 °C, respectively.

### 3.2. Analysis of structures

**Fig. 3:**
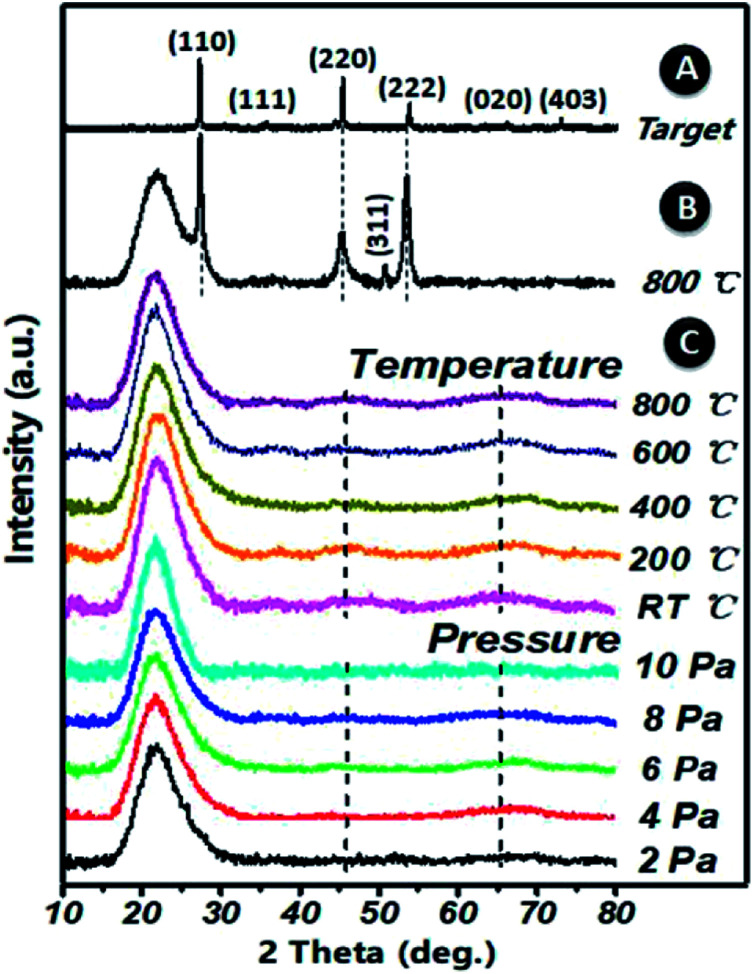
An XRD pattern of the (Ga_2_O_3_)_0.1_(Co)_0.5_(ZnSe)_0.4_ target for PLD in region A. An XRD pattern of (Co)_0.1_(ZnSe)_0.9_ thin film at a substrate temperature of 800 °C grown on a quartz substrate in region B. XRD patterns of (Ga_2_O_3_)_0.1_(Co)_0.5_(ZnSe)_0.4_ thin film grown on a quartz substrate in region C.

XRD patterns of (Ga_2_O_3_)_0.1_(Co)_0.5_(ZnSe)_0.4_ thin film, the (Ga_2_O_3_)_0.1_(Co)_0.5_(ZnSe)_0.4_ target and (Co)_0.1_(ZnSe)_0.9_ thin film are shown in Fig. 3. The (Ga_2_O_3_)_0.1_(Co)_0.5_(ZnSe)_0.4_ target has six planes at (110), (111), (220), (222), (020) and (403), (110), (220) and (222) correspond to ZnSe (JCPDS 37-1463), (111) corresponds to Co (JCPDS 89-4307), and (020) and (403) correspond to Ga_2_O_3_ (JCPDS 43-1012), respectively. From the acquired pattern of the target, all elements of (Ga_2_O_3_)_0.1_(Co)_0.5_(ZnSe)_0.4_ have been proven to be present before PLD experiments. Meanwhile, the pattern of the single doped material, Co–ZnSe thin film, is also displayed in region B, which was addressed in our previous work prior to investigating multi-doped materials. The crystalline structure of Co singly-doped into ZnSe is confirmed in a high temperature region, such as 800 °C, using XRD, which also proves that the structure of Co–ZnSe is cubic zinc blende. This means that Co has been doped into ZnSe and substituted some of the Zn atoms. Then, the XRD patterns of (Ga_2_O_3_)_0.1_(Co)_0.5_(ZnSe)_0.4_ thin film grown on a quartz substrate are shown in region C. Three wide diffraction peaks are observed at around 22°, 47°, and 65°, which are attributed to the quartz substrate, Zn_0.1_Co_0.5_Ga_2_O_4_, and Ga_2_O_3_, respectively.^37^ The diffraction peak intensity of the quartz substrate is far greater than the thin films because the max survey depth of XRD is less than 10 μm, which is an order of magnitude larger than the thickness of the film. In addition, the minimum survey phase content detected using XRD is more than 5%, according to different material. Therefore, the amorphous structure of (Ga_2_O_3_)_0.1_(Co)_0.5_(ZnSe)_0.4_ thin film is detected using XRD.^38^ What is important is the non-existence of grain boundaries, which is considered as the most attractive feature of an amorphous structure. Compared with Co singly doped into ZnSe, doping with Ga_2_O_3_ is determined to lead to an amorphous structure in the film due to its anionic densely stacked structure. Besides, under preparation conditions of 10 Pa, weak intensity peaks appeared at 47° and 65°, which is consistent with the conclusion of the study on thickness. Furthermore, the average peak values for all films with changes in temperature are greater than those for different degrees of pressure. Thus, in order to further achieve crystal to amorphous transformation under experimental conditions, the substrate temperature must be controlled reasonably.

**Fig. 4:**
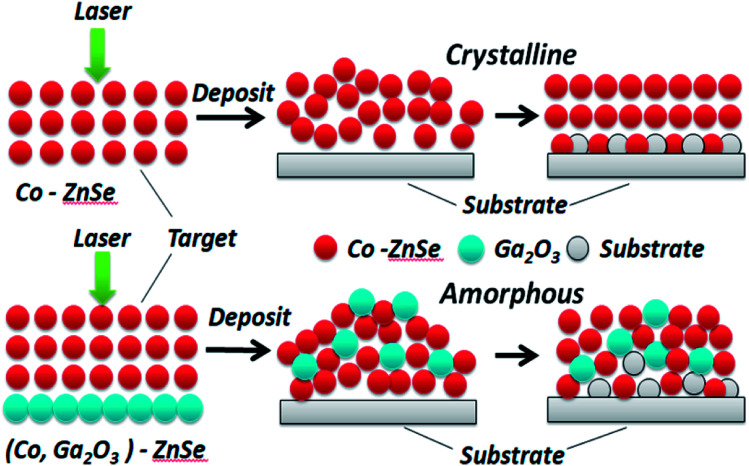
The different structure forming processes for Co and (Co, Ga_2_O_3_) doped ZnSe.

Both crystalline and amorphous materials can be fabricated *via* the method of PLD. The different structure forming processes for Co and (Co, Ga_2_O_3_) doped ZnSe are shown in Fig. 4. Molecules or atoms of Co–ZnSe, Ga_2_O_3_ and the substrate are represented by red, blue and gray balls. From the XRD patterns, we know that the thin film source material has a crystalline structure, which is used as the target in PLD for depositing the thin films. In terms of Co–ZnSe, an irregular arrangement is presented by the atoms or molecules in the process of deposition. The XRD pattern of Co–ZnSe shows that the Co atoms have entered into the ZnSe structure. So the attraction between the atoms and the sizes are similar to one another. Besides, the activities of the atoms or molecule can be improved though changing the preparation conditions. Thus, the atoms tend to form a regular arrangement and a crystalline structure in this situation. Besides, if we consider an interface between two or more different materials, the important feature of abruptness can't be ignored. A feature might be sharp and abrupt on an atomic scale or it might be “washed out” through interdiffusion or the formation of new chemical compounds. The compounds do not affect the structure of the material and are also arranged in a regular arrangement.

But for (Co, Ga_2_O_3_)–ZnSe, the situation is changed. The interactions between the atoms of the material have changed, obviously because of the incorporation of Ga_2_O_3_ molecules. Besides, after the addition of Ga_2_O_3_, the size differences between the atoms are too large, hindering the formation of a crystal structure. Then, substrate atoms might enter into the structure of a material through interdiffusion, but they can't develop new chemical compounds at the interfaces. Finally, an amorphous structure is formed. We do not seek to crystallize this material, because amorphous materials have many advantages, such as being very suitable for flexible displays, solar cells and glass. What's more, our materials belong to the chalcogenide material class. Traditional chalcogenide glasses have not progressed in relation to doping and the matching of materials and scale, but thin films with glassy states have been achieved on the nano-scale in this work.

### 3.3. The optical properties

Transmission spectra of (Ga_2_O_3_)_0.1_(Co)_0.5_(ZnSe)_0.4_ thin film at different substrate temperatures and pressures are shown in Fig. 6. As the pressure rises, more systematic changes are described in Fig. 6(a) and (b), which refer to the transmittance from 55% to 88% and 64% to 92%, respectively. Comparing Fig. 6(a) with Fig. 6(b), the transmittance of thin film on a sapphire substrate is slightly higher than that on a quartz substrate under the same preparation conditions of pressure, but the better absorption abilities of thin film on a quartz substrate are still reflected. Comparing Fig. 6(c) with Fig. 6(d), there is not a bit difference in the range of transmittance between thin film on quartz (68–98%) and sapphire (70–102%) substrates, but thin film on a quartz substrate still has better absorption than on sapphire. The transmittance at RT °C and 800 °C is higher than at several other temperatures, and almost reaches 100%. Furthermore, comparing Fig. 6(a) with Fig. 6(c), transmittance under conditions of changing temperature is obviously higher than under changing pressure, which is also applicable to Fig. 6(b) and (d). In summary, the existence of high transmittance in the range of 500–3000 nm (visible to mid-infrared) is supported by the transmission spectra. The property of high transmittance is an important index for thin films and one of the necessary conditions for display devices. What's more, under the same preparation conditions, similar transmission properties are exhibited on sapphire and quartz substrates. However, on the same substrate, better transmission properties are produced under preparation conditions of changing temperature rather than pressure. Temperatures of RT °C and 800 °C, and pressure of 10 Pa can be deemed as the optimal preparation conditions for transmission spectra tests.

**Fig. 5:**
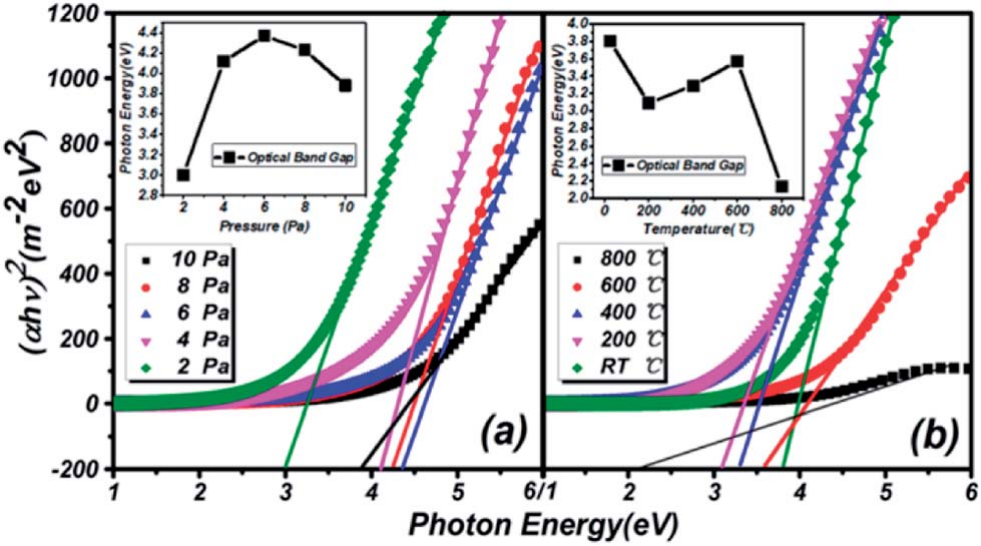
Estimating the optical band gaps of thin films under different conditions on a quartz substrate, based on Tauc plot mapping. The inset shows the relationship between the optical band gap and the different conditions.

**Fig. 6:**
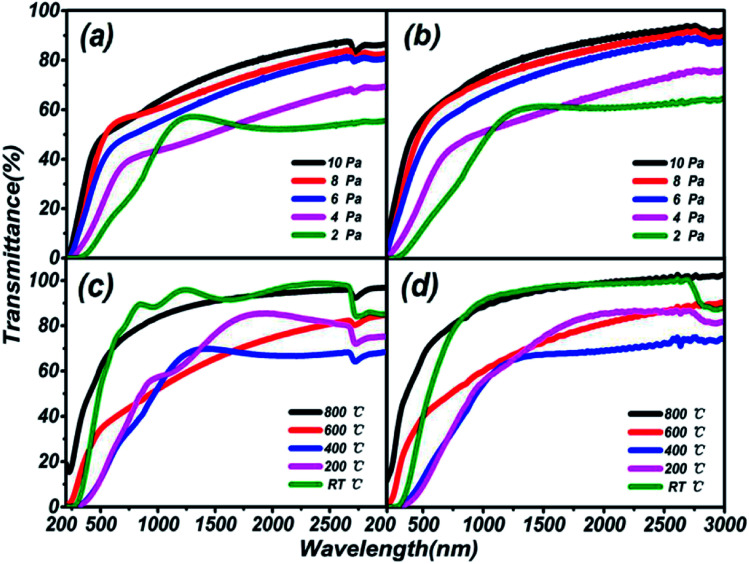
Transmission spectra of (Ga_2_O_3_)_0.1_(Co)_0.5_(ZnSe)_0.4_ thin film on different substrates under preparation conditions of varying temperature and pressure. (a) Transmission spectra of (Ga_2_O_3_)_0.1_(Co)_0.5_(ZnSe)_0.4_ thin film on a quartz substrate under varying pressure. (b) Transmission spectra of (Ga_2_O_3_)_0.1_(Co)_0.5_(ZnSe)_0.4_ thin film on a sapphire substrate under varying pressure. (c) Transmission spectra of (Ga_2_O_3_)_0.1_(Co)_0.5_(ZnSe)_0.4_ thin film on a quartz substrate under varying substrate temperatures. (d) Transmission spectra of (Ga_2_O_3_)_0.1_(Co)_0.5_(ZnSe)_0.4_ thin film on a sapphire substrate under varying substrate temperatures.

The estimation of the optical band gaps of thin films under different conditions on a quartz substrate, based on Tauc plot mapping, is shown in Fig. 5. The absorption coefficient *α* and optical band gap *E*_g_ are related through the Tauc relation:^39^

1 (*αhν*)^2^ = *B*(*hν* − *E*_g_)

where *h* is Planck's constant, *B* is the energy-independent constant, and *ν* is the frequency of the incident photon. The estimated optical band gaps under different preparation conditions of pressure and temperature are illustrated in Table 1. The optical band gap trends with different pressures and substrate temperatures are depicted in the inset of Fig. 5. The reason for the changes in the optical band gaps is the Moss–Burstein effect. The band gap is changed when there is more doping in semiconductors. The unoccupied energy states at the top of the valence band and the middle of the conduction band are separated. N type doping materials make the band gap enlarge and change due to the Fermi level of the conduction band (P type relates to the valence band).

**Table 1:** Optical band gaps of thin films on a quartz substrate

Pressure (Pa)/temperature (°C)	2/RT	4/200	6/400	8/600	10/800
Band gap with different pressure (eV)	3.00	4.12	4.37	4.23	3.88
Band gap with different temperature (eV)	3.80	3.09	3.29	3.57	2.14

The similar trends in optical band gap changes in thin films prepared under two different conditions are reflected in the inset of Fig. 5. Before a pressure of 6 Pa, the optical band gap continually increases, but the opposite trend appears after 6 Pa. In the low pressure region, the particle size in the thin films is continuously refined and reaches a minimum at 6 Pa, which is consistent with the AFM results. According to quantum confinement effects, this situation will directly lead to an increase of the optical band gap of materials. As the pressure continues to rise, the particles will be saturated to a certain extent, and the particles and plume will be impacted by the Ar atmosphere and impurity particles in the vacuum chamber. This phenomenon will lead to the stacking of two thin particles and increase the overall particle size, so the optical band gap will be reduced. Besides, optical thin films with different substrate temperatures are not affected by the preparation conditions at room temperature. Thus, the overall optical band gap is increased by the original Ga_2_O_3_ (4.9 eV) molecule. When the temperature rises to 400 °C or 500 °C, half the temperature of the ZnSe melting point (1100 °C), the force of Co–ZnSe interactions are weakened, and the doping structure is constantly destroyed. So a number of pure ZnSe structures reappear, while the overall optical band gap reduces to near that of pure ZnSe (2.7 eV). However, the film is still amorphous because of the existence of Ga_2_O_3_. What is important is that a kind of doping material with an adjustable band gap from 2.14 to 4.37 eV is realized in this experiment.

**Fig. 7:**
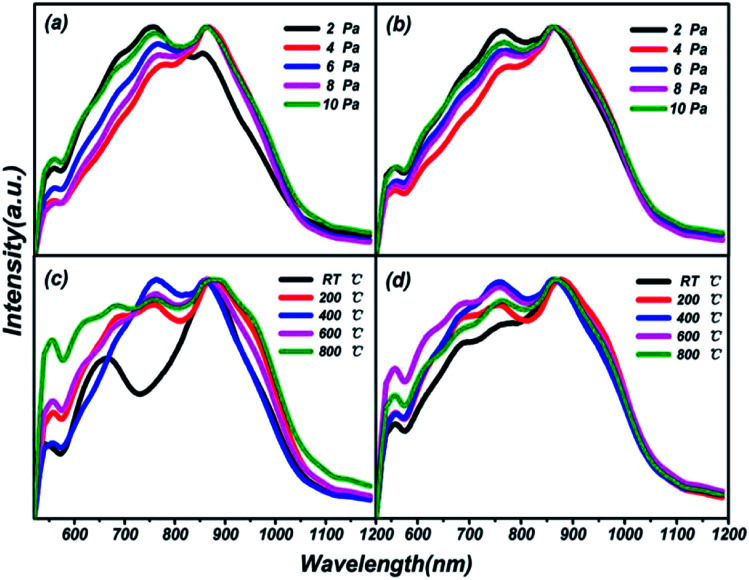
Photoluminescence (PL) spectra of (Ga_2_O_3_)_0.1_(Co)_0.5_(ZnSe)_0.4_ thin film on different substrates under preparation conditions of varying temperature and pressure. (a) Photoluminescence spectra of (Ga_2_O_3_)_0.1_(Co)_0.5_(ZnSe)_0.4_ thin film grown on a quartz substrate prepared at different pressures. (b) Photoluminescence spectra of (Ga_2_O_3_)_0.1_(Co)_0.5_(ZnSe)_0.4_ thin film grown on a sapphire substrate prepared at different pressures. (c) Photoluminescence spectra of (Ga_2_O_3_)_0.1_(Co)_0.5_(ZnSe)_0.4_ thin film grown on a quartz substrate prepared at different substrate temperatures. (d) Photoluminescence spectra of (Ga_2_O_3_)_0.1_(Co)_0.5_(ZnSe)_0.4_ thin film grown on a sapphire substrate prepared at different substrate temperatures.

Photoluminescence (PL) spectra of thin films on different substrates under preparation conditions of varying pressures and substrate temperatures are shown in Fig. 7. Three luminescent peaks from the films, at 557.76 nm, 755.84 nm and 860.36 nm with a 400 nm excitation wavelength, are manifested in the visible photoluminescence spectra shown. The growth processes of the films over all different preparation conditions and substrates are illustrated through the consistencies of the same luminescent centers. In particular, a peak at 660 nm, as a single luminescent center, is found at RT in Fig. 7(c), which is attributed to the particularity of the structure of the thin film at normal temperature compared with at high temperatures. As early as 1975, the characteristics of PL spectra of sulfur-based amorphous semiconductors had been reported by R. A. Street.^40^ No luminescence spectra corresponding to the absorption spectra of chalcogenide amorphous semiconductors have reported the first feature. Meanwhile, the PL spectra of all sulphur system materials show not a very sharp peak but a larger half width, which is the second feature mentioned. The results of our research are in good agreement with the conclusions of R. A. Street. The phenomena can be explained *via* the strong electron-lattice coupling.^41^ The number of luminescence peaks from films prepared at different temperatures is more than those at different pressures, which means that energy transfer between different energy levels is more frequent, and easier to activate. Good optical properties in the visible and near infrared range in thin film of (Ga_2_O_3_)_0.1_(Co)_0.5_(ZnSe)_0.4_ have been elicited.

### 3.4. The electromagnetic properties

**Fig. 8:**
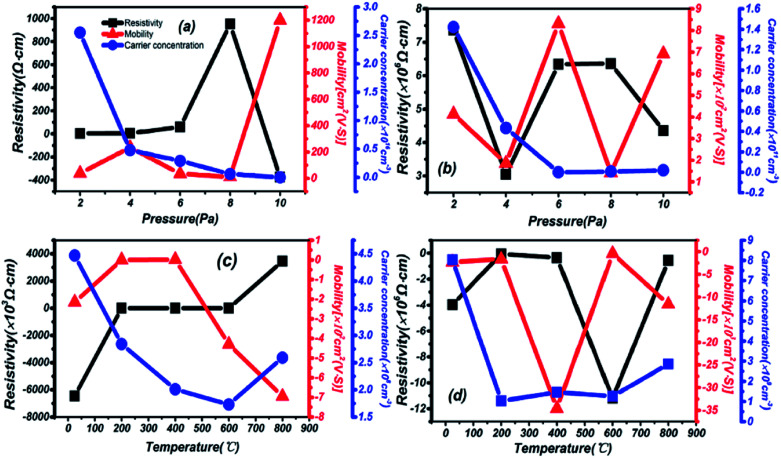
The dependence of Hall mobility, carrier concentration and resistivity on different preparation conditions. (a) Thin films on a quartz substrate at different pressures. (b) Thin films on a sapphire substrate at different pressures. (c) Thin films on a quartz substrate at different temperatures. (d) Thin films on a sapphire substrate at different temperatures.

The dependencies of Hall mobility, carrier concentration and resistivity on different preparation conditions are shown in Fig. 8. High carrier concentration and low resistivity are depicted in thin films grown on a quartz substrate at different pressures in Fig. 8(a). The carrier concentration reaches 10^16^, and the resistivity is in a relatively low state in the range of 2–6 Pa. Then, the sharply increasing resistivity is exemplified in films grown on a sapphire substrate in Fig. 8(b), 3 orders of magnitude higher than on quartz. Meanwhile, there is a mobility increase on a different substrate of 10^2^ and the property of oscillation, especially at 6 Pa: the highest for sapphire. Besides, negative resistivity and mobility are observed in Fig. 8(c) and (d) due to changes in the carrier migration direction, resulting from a different installation direction of the electrode during testing. But the correctness and analysis of the results are not affected by this phenomenon. An even lower carrier concentration is presented by thin films grown on a quartz substrate at different temperatures in Fig. 8(c), but the resistivity is also reduced at the same mobility compared to Fig. 8(b). A lower carrier concentration is also found in Fig. 8(d) for thin films grown on a sapphire substrate, and the highest mobility appears under temperature conditions of 400 °C.

In summary, a strong dependence on the preparation conditions and substrate during the process of deposition is reflected in the resistivity, carrier concentration and mobility of thin films. Under the same preparation conditions, a lower resistivity and higher carrier concentration are presented on a quartz substrate. Then, on the same substrate, better electrical and semiconductor performance are reflected under changing pressure and temperature conditions, mainly in the mid-temperature (200–600 °C) and high-pressure (6–10 Pa) areas. From the overall results, 2.55 × 10^16^ cm^−3^, 3.46 × 10^4^ cm^2^ (V s)^−1^ and 4.52 Ω cm correspond to the highest carrier concentration, highest mobility and lowest resistivity, respectively. Compared with similar materials in other reports, such as 26.70 cm^2^ (V s)^−1^ for IGZO,^42^ the higher mobility in this material has been proved through Hall effect measurements.

### 3.5. The surface morphologies

**Fig. 9:**
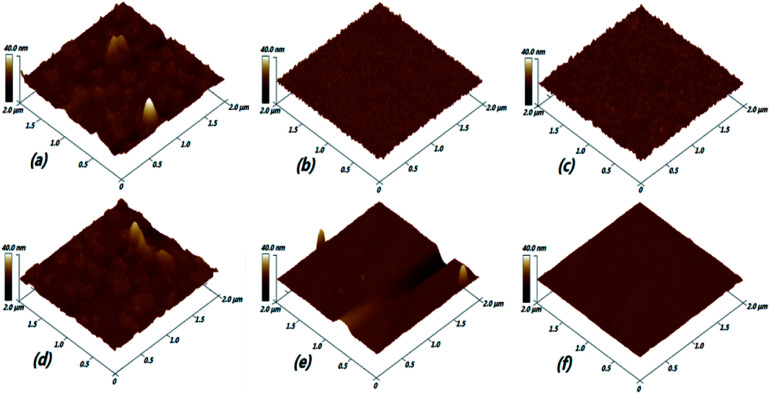
AFM images of (Ga_2_O_3_)_0.1_(Co)_0.5_(ZnSe)_0.4_ thin films prepared at various pressures and substrate temperatures: (a) 2 Pa; (b) 6 Pa; (c) 10 Pa; (d) RT; (e) 400 °C; and (f) 800 °C.

AFM images of (Ga_2_O_3_)_0.1_(Co)_0.5_(ZnSe)_0.4_ thin films prepared at various pressures and substrate temperatures are displayed in Fig. 9, with a 2 × 2 μm area. The large particles and flake shapes are obviously shown in the film morphology at 2 Pa in Fig. 9(a). These characteristics lead to low transmittance and a small optical band gap, as also seen under a pressure of 4 Pa, which is consistent with the refractive index results. Then, upon increasing the pressure in the vacuum chamber, the large particles and flake shapes are decomposed into small particles on the surface of the thin films under pressures of 6 Pa and 10 Pa, as seen in Fig. 9(b) and (c). Those small particles are orderly and densely arranged on the surface of the film, and are larger on the film prepared at 10 Pa than at 6 Pa. This result proves the reason for the increase in the optical band gap, especially at a pressure of 6 Pa. In general, the morphology of a film can be greatly influenced by the crystallinity, thickness and grain size. But for amorphous films, there is no concept of crystallinity and grain, so it may be more reasonable to explain this behaviour as follows: with an increase in pressure, the Ar content in the vacuum chamber continuously increases. Then atoms, ions or clusters on the target produced *via* laser sputtering will collide with Ar molecules in the vacuum chamber. The plasma and process of film forming are affected by this force, which will drive particles to combine more densely. As time goes on, their arrangement will also tend to be neat. The surface roughness of films prepared under different pressures is higher than under different substrate temperatures, which directly illustrates the lower average transmittance under conditions of pressure.

There is a great difference between pressure and substrate temperature, except for RT conditions, where there is similar surface morphology to thin films prepared at 2 Pa. But the transmittance of thin film at RT is higher than at 2 Pa, proving that low pressure has great influence on the transmittance of thin films. Upon increasing substrate temperature, the number of large particles is significantly reduced. Low-lying and high valley areas appeared on the surface. It can be considered that the smooth surface of the film has been greatly improved at 400 °C, as shown in Fig. 9(e). In middle temperature regions, such as 400 °C and 600 °C, some low-lying and high valley areas are the main reasons for the decrease in transmittance, because these areas could make some light reflect or scatter and they are thicker. Then, thin films with extremely smooth and bright surface morphology, without large particles and other impurities, are prepared at a temperature of 800 °C. This phenomenon can be explained by the motion of particles. When particles are excited by a laser, including atoms, ions and clusters, they arrive at the substrate surface and cannot stop moving at once. In other words, they can also move over a certain range on the substrate. The particles have low mobility at lower substrate temperatures, so an amorphous structure is finally formed. However, when a higher substrate temperature is used, larger thermodynamic energy and higher mobility can be obtained. Furthermore, large particle structures can be further refined and adsorbed impurity particles can be evaporated under such preparation conditions. Therefore, the important effects of high temperature preparation conditions on the film are demonstrated. From the results of AFM, the effects of different preparation conditions on the optical properties of thin films have a certain degree of correlation with the surface morphology, such as transmittance, optical band gap and refractive index. But surface morphology is not very related to the photoluminescence properties of materials.

## 4. Conclusions

In this work, we examined the effects of different temperatures, pressures and substrates on deposited thin films of (Ga_2_O_3_)_0.1_(Co)_0.5_(ZnS/Se)_0.4_, fabricated *via* a PLD method. Thin films in a glassy state have been achieved on the nano-scale in this experiment. A stable vacuum chamber atmosphere with fewer gaseous impurities during the process of preparation was detected using a mass spectrometer. The thicknesses of the thin films are greatly affected by the preparation conditions. The poor light transmittance of thin films prepared at 4 Pa and 600 °C is revealed through the results of refractive index measurements. The amorphous structure is confirmed *via* XRD. The optimum preparation conditions are reflected in the transmission spectra, especially conditions of RT, 800 °C and 10 Pa. Meanwhile, the optical band gaps of materials prepared under changing pressure conditions are larger than those under changing temperature conditions, with band gaps calculated based on Tauc plot mapping. More frequent and easier activated energy transfer between energy levels occurs under conditions of changing temperature, as indicated in the PL spectra. Lower resistivity and a higher carrier concentration on a quartz substrate are shown in the results of Hall effect tests, and better electrical and semiconductor performances are reflected upon changing the pressure and substrate temperature. The important effects of high temperature preparation conditions on the films are demonstrated *via* AFM. Therefore, the main properties of the films are strongly influenced by changing the substrate temperature. 800 °C in the high temperature zone and 400 °C in the middle temperature zone should be chosen as optimum substrate temperatures. The high pressure area of 6–10 Pa can be also deemed as optimal preparation conditions. In summary, high transmission in the middle infrared range, good optical properties in the visible and near infrared range, higher mobility and better electrical properties are demonstrated in amorphous semiconductor thin films of (Ga_2_O_3_)_0.1_(Co)_0.5_(ZnSe)_0.4_. In our next work, we will further study the application of this material to solar cells, field effect devices, threshold switches and memory switching devices. Meanwhile, we will try to fabricate chalcogenide glass using the material with a glassy state. Besides, we will investigate the properties of this material by changing the proportions under the same experimental conditions.

## Conflicts of interest

There are no conflicts to declare.

## Supplementary Material
